# A semifluorinated alkane (F4H5) as novel carrier for cyclosporine A: a promising therapeutic and prophylactic option for topical treatment of dry eye

**DOI:** 10.1007/s00417-016-3572-y

**Published:** 2017-01-14

**Authors:** Uta Gehlsen, Tobias Braun, Maria Notara, Sonja Krösser, Philipp Steven

**Affiliations:** 1 Department of Ophthalmology, Medical Faculty, University of Cologne, Kerpenerstrasse 62, 50937 Cologne, Germany; 2 Cluster of Excellence: Cellular Stress Response in Aging-Associated Diseases (CECAD), University Cologne, Cologne, Germany; 3 Novaliq GmbH, Heidelberg, Germany

**Keywords:** Dry-eye disease, Cyclosporine, Desiccating stress, Mouse model

## Abstract

**Purpose:**

Cyclosporine A (Cs) has been used as effective topical therapy for inflammatory dry eye disease since more than a decade. However, due to its lipophilic character, Cs is formulated as emulsions or oily solutions for topical application. This experimental study aimed to test if the use of semifluorinated alkanes (SFAs) as a preservative-free, well-tolerated non-stinging or burning vehicle maintains or even improves the benefits of Cs in the topical therapy of dry-eye disease.

**Methods:**

Desiccating stress was applied to C57BL/6 mice for 14 consecutive days to induce experimental dry-eye. Cs dissolved in SFA (perfluorobutylpentane = F4H5with 0.5% Ethanol), F4H5 with 0.5% ethanol only, 0.05% Cs (Restasis®), and dexamethasone (Monodex®) were applied three times daily beginning either at day 4 or day 11 of desiccating stress for up to 3 weeks after end of dry-eye induction.

**Results:**

In comparison to other groups, Cs/F4H5 demonstrated high efficacy and earlier reduction of corneal staining. In this study, Cs/F4H5 had the ability to maintain conjunctival goblet cell density once applied on day 4. Flow cytometry analysis from cervical lymphnodes demonstrated a significantly lower CD4+ and CD8+ T-cells in the Cs/F4H5 group following 3 weeks of therapy than at baseline, but no difference in regulatory T cells from regional lymphnodes were seen.

**Conclusions:**

Overall, compared to a commercially available Cs formulation (Restasis®) and dexamethasone, Cs/F4H5 was shown to be equally effective but with a significantly faster therapeutic response in reducing signs of dry-eye disease in an experimental mouse model.

## Introduction

Dry-eye disease (DED) is one of the most common disorders of the ocular surface, associated with dysfunction of the lacrimal functional unit, changes in tear fluid, corneal and conjunctival epitheliopathy, and consecutive inflammation [1, 2]. Lighter cases of DED and consecutive ocular discomfort are mainly managed with artificial tears, while therapeutic treatment of more severe and chronic cases of dry eye and underlying inflammation include topical steroids or cyclosporine (Cs), topical or oral antibiotics, topical autologous serum drops, and even systemic immunosupressives. However, some of these therapeutic strategies cause a wide range of side-effects, e.g., cataract, glaucoma, or infections, but also a strong burning sensation during topical application [3, 4]. With regard to the use of immunosuppressives, currently the only FDA-approved (U.S. Food and Drug Administration) medication for dry-eye disease is a 0.05% cyclosporine emulsion (Restasis®, Allergan Inc., Irvine, CA, USA), whereas in Europe 0.1% cyclosporine has recently been approved by the EMA (European Medicines Agency) for severe keratitis in DED (Ikervis®, Santen).

Cyclosporine is a calcineurin inhibitor, targeting specifically the T-cell response, and was described to increase tear secretion, decrease epithelial damages, increase goblet cell density and visual acuity, but also to improve subjective symptoms in dry-eye patients [5–7]. However, in many countries Restasis® or Ikervis® are not available or restricted to only severe cases, and alternatively Cs eye drops have to be compounded by pharmacies using several non-standardized formulations. Furthermore, as the lipophilic Cs has to be formulated using oils and/or surfactants, e.g., castor oil or polysorbate 80, this often leads to intolerance, burning sensation, or visual disturbance. Therefore, application is frequently discontinued [4, 8]. As an alternative to existing formulations semifluorinated alkanes (SFAs) were introduced as a new delivery platform, enabling a simple and preservative-free formulation of Cs.

SFAs (e.g., perfluorobutylpentane = F4H5) are linear molecules composed of a hydrocarbon and a perfluorocarbon segment holding special features such as a certain degree of lipophilicity, low surface and interface tension, and high biocompatibility. They have the potential to dissolve water-insoluble substances, e.g., the lipophilic Cs [9, 10]. Using an ex-vivo eye irritation test (EVEIT) it was previously shown that the SFAs F4H5 and F6H8 are well tolerated and cause no toxic effects on enucleated rabbit corneas [11]. Also, a recently conducted post-marketing surveillance study using F6H8 as artificial tears demonstrated the safety and tolerability of SFAs in clinical treatment of hyperevaporative DED [12]. F6H8 is now marketed as EvoTears® (Ursapharm Arzneimittel GmbH, Saarbruecken, Germany) in Germany and Switzerland.

In this study, a mouse model of experimental dry eye disease was used to investigate the effect of the semifluorinated alkane F4H5 as a novel carrier for Cs as topical treatment for DED during early and late therapeutic applications.

## Materials and methods

### Induction of dry eye

Experimental dry eye (EDE) was induced in 10–12-week-old female C57BL/6 mice purchased from Charles River (Sulzfeld, Germany) as previously published [13]. Mice were placed in a controlled environment chamber (humidity 30 ± 5%, constant airflow, temperature 25 ± 1 °C) for 14 days. Scopolamine was administered (0.1 mg/day) by subcutaneous implanted osmotic pumps (Alzet, model #1002). Pumps were explanted after 2 weeks (day 14). After 14 days of desiccating stress, animals were transferred to normal controlled housing conditions (humidity 45–55%, no airflow, temperature 24 ± 2 °C) for another 3 weeks. Climatic changes were hourly logged and checked automatically (KlimaLogg-Pro, TFA Dostmann, Germany).

All animals were treated according to the German Animal Protection Law (LANUV), the local regulations of the University of Cologne and the ARVO statement for the use of animals in ophthalmic research.

### Topical therapy

**Fig. 1:**
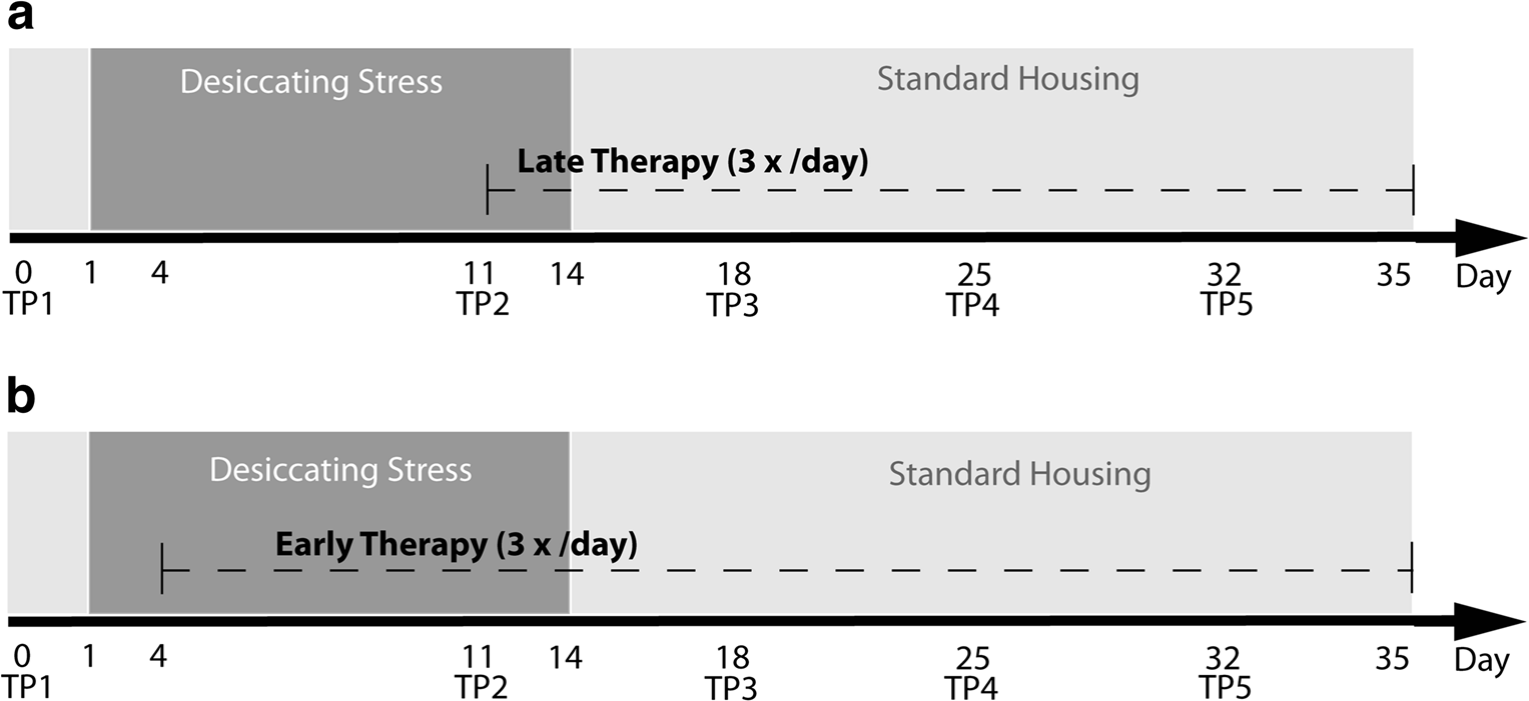
Experimental set-up. **a** Late therapy regimen (therapeutic), **b** Early therapy regimen (prophylactic): TP1 to TP5—time points for clinical scoring (tear production, corneal staining)

Two different therapeutic regimens were applied: Topical therapy (5 μl/eye, 3 times daily) was applied from day 11 (late therapy/therapeutic) or from day 4 (early therapy/prophylactic) of experimental dry eye (Fig. 1a, b). Mice were distributed in four groups: (1) 0.05% Cs/F4H5 with 0.5% ethanol as co-solvent (Novaliq GmbH, Heidelberg, Germany), (2) carrier F4H5 with 0.5% ethanol (Novaliq GmbH, Heidelberg, Germany), (3) Restasis® (Allergan Inc., Irvine, CA, USA) and (4) unpreserved Dexamethasone (Monodex®1 mg/ml, TheaPharma, Berlin, Germany). A control group was left untreated and received no eye drops, but was housed under the same desiccating stress and standard housing conditions as the four therapy groups.

### Readout parameters

**Fig. 2:**
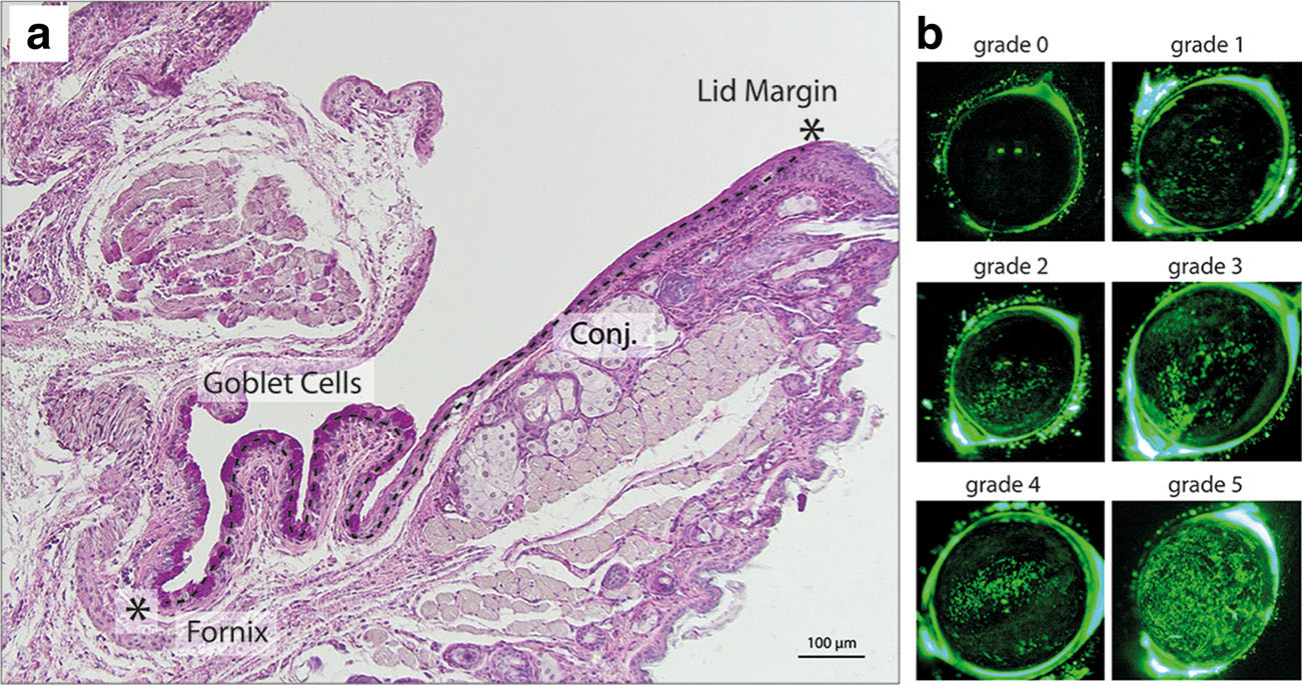
**a** Bright-field microscopy of paraffin embedded section of lower lid with attached conjunctiva (*Conj.*) PAS-staining. Conjunctival goblet cells (*bright pink*) are densely distributed in the conjunctiva in close proximity to the fornix. Goblet cell counts were performed from the lid margin to fornix (indicated by *asterisks and dotted line*). **b** Representative in-vivo images from fluoresceine staining of the murine cornea. Epithelial damage is stained in *green*. Grading ranged from 0 (no staining) to 5 (surface almost entirely covered with stained spots)

Clinical signs of dry eye )production of tear fluid and corneal epitheliopathy) were measured once a week as folllows: time point [TP] 1: baseline-day 0, TP 2: day 11, TP 3: day 18, TP 4: day 25, TP 5: day 32 (Fig. 1a and b). For measurement of tear production, phenol red threads (Zone Quick Thread, Oasis Medical, USA) were placed into the inferior cul-de-sac for 30 s and recorded in millimeters. Corneal damage was detected by fluoresceine staining: 5% fluoresceine in normal saline solution was applied to the eye, carefully wiped off after 30 s and graded under blue light using a modified Oxford grading scheme with severities ranging from grade 0 to grade 5 (Fig. 2a) [14].

At day 35, all mice were sacrificed and eyes including conjunctiva were removed. For quantification of goblet cells the lower lid was paraffin-embedded and sectioned, and goblet cells were stained with PAS (periodic acid-Schiff) dye. Images were taken using a brightfield microscope (Olympus BX53; Olympus Deutschland GmbH, Hamburg, Germany) and a color camera (Olympus UC10, Olympus Deutschland GmbH, Hamburg, Germany). Goblet cells were counted manually from the lid border to the fornix, and stated as cells/100 μm using ImageJ Software (National Institutes of Health, Bethesda, MD, USA). One representative slide out of the central region of the conjunctiva was analyzed from seven to 12 eyes/group depending on the availability of exactly aligned cross-sections (Fig. 2b).

### Flow cytometry analysis (FACS)

FACS analyses were performed in one experiment following the late therapeutic regimen. Draining lymphnodes of three control mice and three mice receiving F4H5 or Cs/F4H5 were collected at TP1, TP3 and TP5. For T-cell and regulatory T-cell (Treg) analysis, single cell suspensions were stained with FITC-conjugated anti-CD8, APC-conjugated anti-CD4, PE-conjugated anti-CD25 (all Biolegend, San Diego, CA, USA) and a FITC-conjugated anti-FoxP3 (BD Biosciences, Heidelberg, Germany) antibody according to the manufacturers’ instructions. Stained samples were examined on a Guava easyCyte™ HT (Merck Millipore, Darmstadt, Germany), and analyzed using FlowJo Software (FlowJo LLC, Tree Star Inbc., Ashland, OR, USA).

### Statistical analysis

Results were presented as mean ± SD of *n* = 10 eyes of five mice in each experiment (FACS analysis, three mice/group). All experiments were performed two times; data presented here are unpooled from a single experiment. Since all data were positively tested for a Gaussian distribution (Kolmogorov–Smirnov test), the statistical analysis were performed by univariate ANOVA with post hoc test (LSD) using SPSS (Software version 21, IBM). *P*-values of *p* ≤ 0.05 were considered to be significant.

## Results

### Late therapy regimen

#### Tear production

**Fig. 3:**
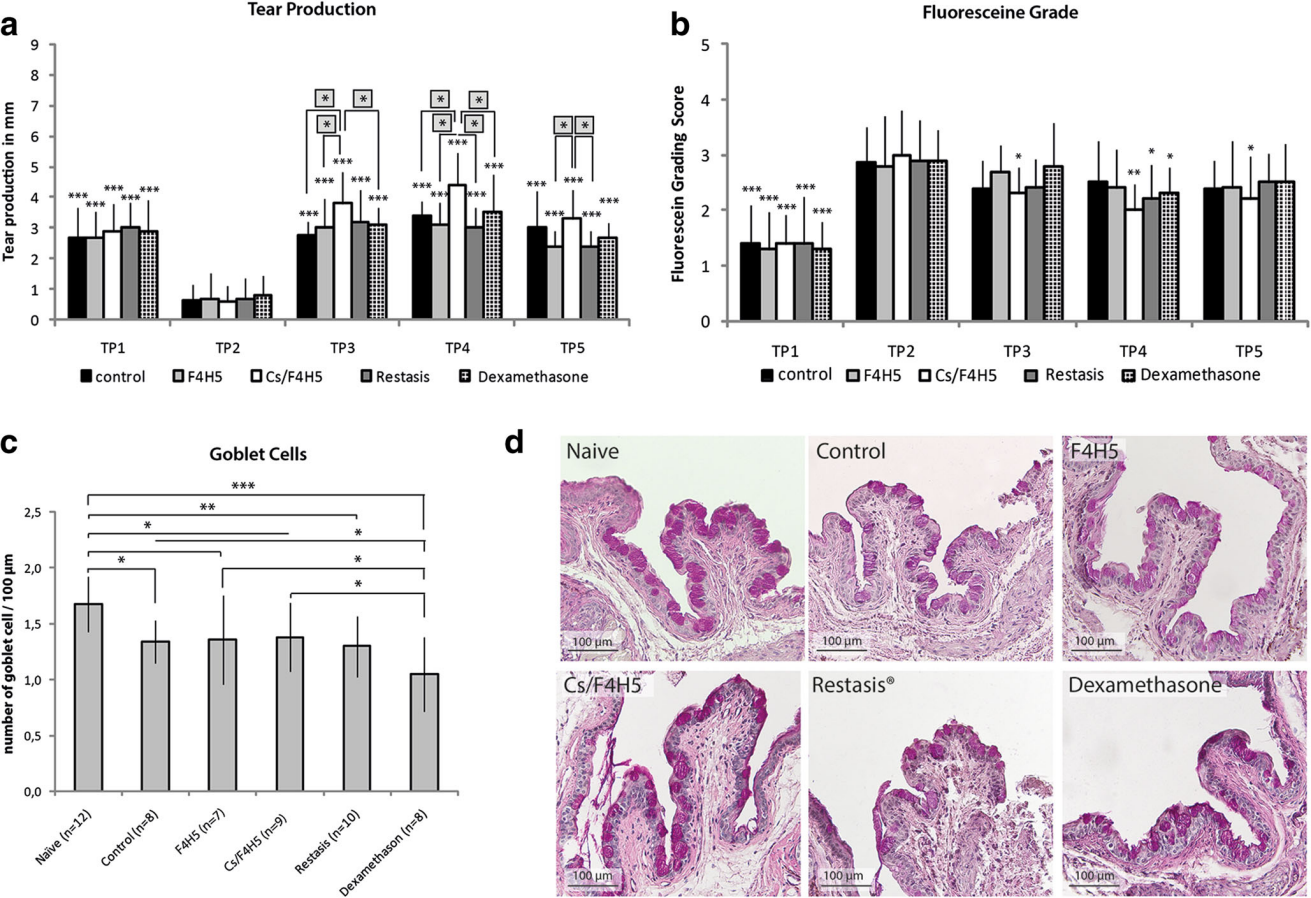
Tear production, fluoresceine staining, and goblet cell density under late therapy regimen before (TP1: baseline), during EDE (TP2) and a following topical treatment (TP3–TP5) of EDE: **a** Tear production: data represent the tear production in mm of each group as mean ± SD (*n* = 10 eyes/group). Late therapy with Cs/F4H5 led to a higher increase of tear production compared between groups at every time point. A comparative group analysis comparing the differences between groups was performed at every evaluation time point (*asterisks in grey squares placed above*). **b** Fluoresceine staining: data are representing the fluoresceine staining score each group as mean ± SD (*n* = 10 eyes/group) Late therapeutic treatment with Cs/F4H5 led to a significant earlier improvement of epithelial staining at TP3. **c** Goblet cell density: all groups showed decreased GC density compared to naïve mice. Treatment with dexamethasone resulted in a lower number of GC compared to F4H5, Cs/F4H5, and control group (mean ± SD, *n* = number of investigated eyes). *P*-values ≤ 0.05 were considered to be significant (* *p* ≤ 0.05, ** *p* ≤ 0.001, *** *p* ≤ 0.0001). Significances refer to TP2 (**a** + **b**). **d** Representative images of conjunctival goblet cell distribution (PAS-staining) in all treatment groups at TP5. All treatment groups demonstrated reduced goblet cells in comparison to the naïve untreated control

Tear production measured by phenol red threads demonstrated a significant increase of tear production in all groups after termination of desiccating stress and following 7 days of treatment all groups in comparison to TP2. Overall levels of tear production were similar to TP1 prior to EDE induction. Comparative group analysis for TPs 3–5 demonstrated that Cs/F4H5-treated mice had a significantly stronger increase of tear production after EDE compared to F4H5, Restasis®, dexamethasone and the untreated control. At TP5, the effect of Cs/F4H5 compared to the control group was less pronounced, although still measurable compared to F4H5 alone and Restasis® (Fig. 3a).

#### Corneal fluoresceine staining

Analysis of corneal damage using fluoresceine staining following late therapy demonstrated a significant increase of the staining in all groups following EDE at TP2. Following 1 week of therapy (TP3), a significant decrease of the fluoresceine staining was observed only in the Cs/F4H5 group (Fig. 3b). Restasis® and dexamethasone treatment resulted in decreased fluoresceine staining at TP4 at the earliest, whereas the reduction of staining in the Cs/F4H5 group increased further at TP5. Only Cs/F4H5 demonstrated a remaining significant decrease of corneal staining in comparison to TP2 (onset of therapy).

#### Goblet cell density

In the late therapeutic regimen, naïve mice demonstrated a significant higher goblet cell (GC) density at TP5 compared to all groups (Fig. 3c + d). Dexamethasone-treated mice had a significant lower number of GC compared to all other groups after late therapy. Cs/F4H5-treated mice also demonstrated a significant lower goblet cell count than the naïve control, but no difference to any treatment group except dexamethasone.

### Early therapy regimen

#### Tear production

**Fig. 4:**
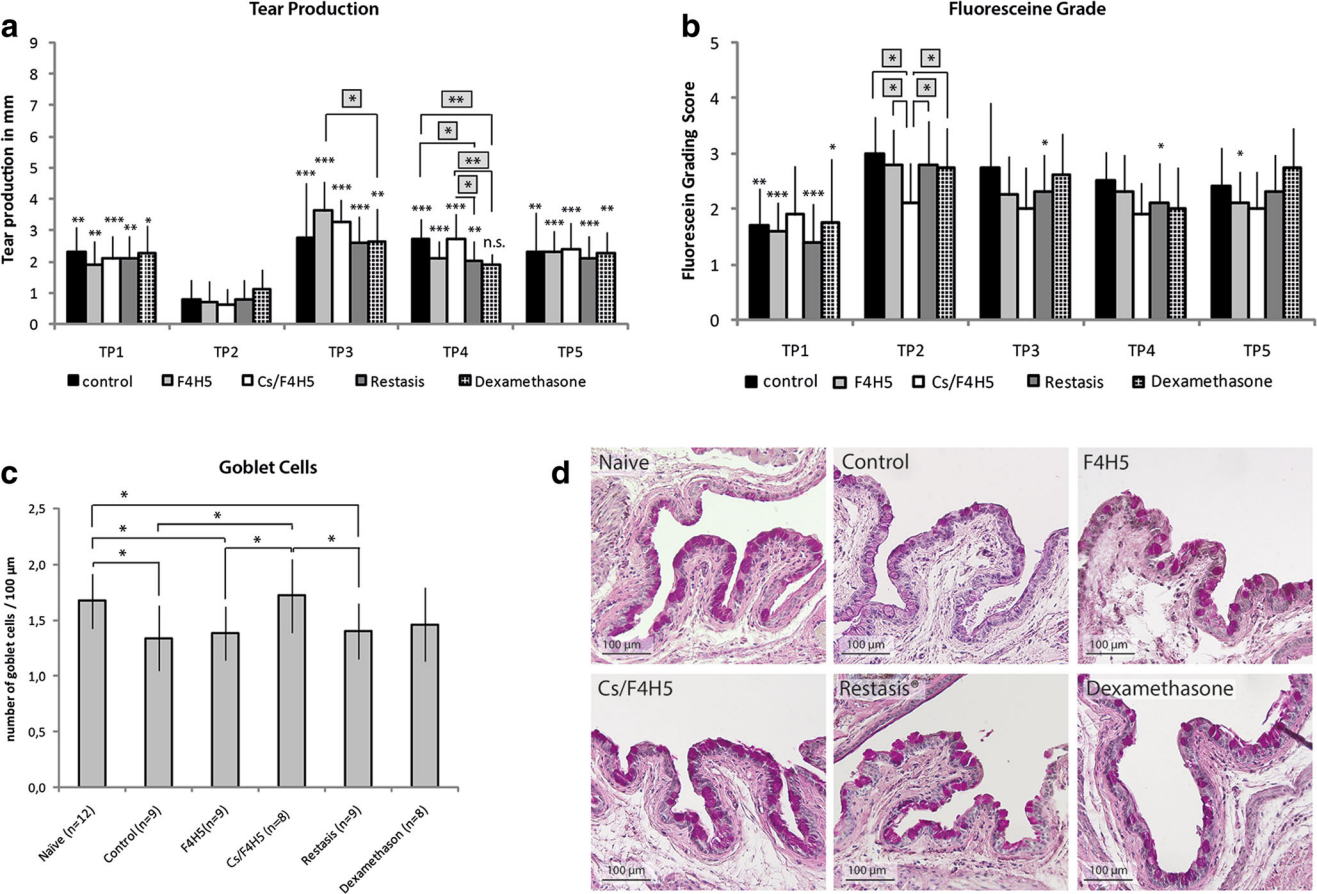
Early therapy regimen **a** Tear production before (TP1: baseline), during EDE (TP2) and a following topical treatment (TP3–TP5) of EDE: data are representing the tear production in mm of each group as mean ± SD (*n* = 10 eyes/group). **b** Fluorescein staining grade before (TP1-baseline), during EDE (TP2) and after a following topical treatment (TP3–TP5) of EDE: Data are representing the fluorescein grading score of each group as mean ± SD (*n* = 10 eyes/group). Early therapy resulted in significant less epithelial staining in the Cs/F4H5 group already at TP2. **c** Expression of goblet cells. After early treatment with Cs/F4H5 GC density remained comparable to naïve mice, whereas in untreated control, F4H5, Restasis® and Dexamethasone number of GC was decreased. *P*-values ≤ 0.05 were considered to be significant (* *p* ≤ 0.05, ** *p* ≤ 0.001, *** *p* ≤ 0.0001). Significances refer to TP2 (**a** + **b**). A comparative group analysis (**a** and Fig. 3b) comparing the differences between groups at every time point was performed, results are placed above (*asterisks in grey squares*) evaluating. **d** Representative images of conjunctival goblet cell distribution (PAS-staining) in all treatment groups at TP5. Only in the group treated with Cs/F4H5 no goblet cell loss was visible

In the early treatment regimen, all groups demonstrated a significant decrease of tear production after EDE and 1 week of concomitant therapy at TP2 compared to TP1. Thereafter, at TP3 and 2 weeks of concomitant application of drugs and carrier, tear production increased again significantly. Comparative group analysis at TP3 demonstrated that tear production was significantly greater in mice receiving F4H5 in comparison to dexamethasone. At TP4, mice receiving Cs/F4H5 for 3 consecutive weeks had significantly higher tear production than the Restasis® and dexamethasone groups. At TP5, no differences between all groups were present, and tear production levels were comparable to levels at TP1 (Fig. 4a).

#### Corneal fluoresceine staining

At TP2 following 2 weeks of desiccating stress and 1 week of concomitant therapy all groups, except the Cs/F4H5 group, demonstrated a significant increase of corneal fluoresceine staining. The between group comparison revealed that corneal staining was significantly lower in this group compared to all other groups. At TP3 and TP4 only Restasis® demonstrated a significant decrease of corneal staining compared to TP2, at TP5 only F4H5 had a significant reduced corneal staining in comparison to TP2. In the Cs/F4H5 group no change of corneal staining in comparison to baseline levels at TP1 at any time point was detectable (Fig. 4b).

#### Goblet cell density

Analysis of the goblet cell (GC) density after early therapy in the bulbar and palpebral conjunctiva of the lower lid resulted in preservation of a normal GC density in Cs/F4H5-treated animals (Fig. 4c, d), compared to naïve mice. Untreated controls and groups receiving F4H5 and Restasis® showed a significantly decreased number of GC compared to naïve mice. Mice that received dexamethasone showed a difference neither to naïve nor control mice.

### FACS analysis

**Fig. 5:**
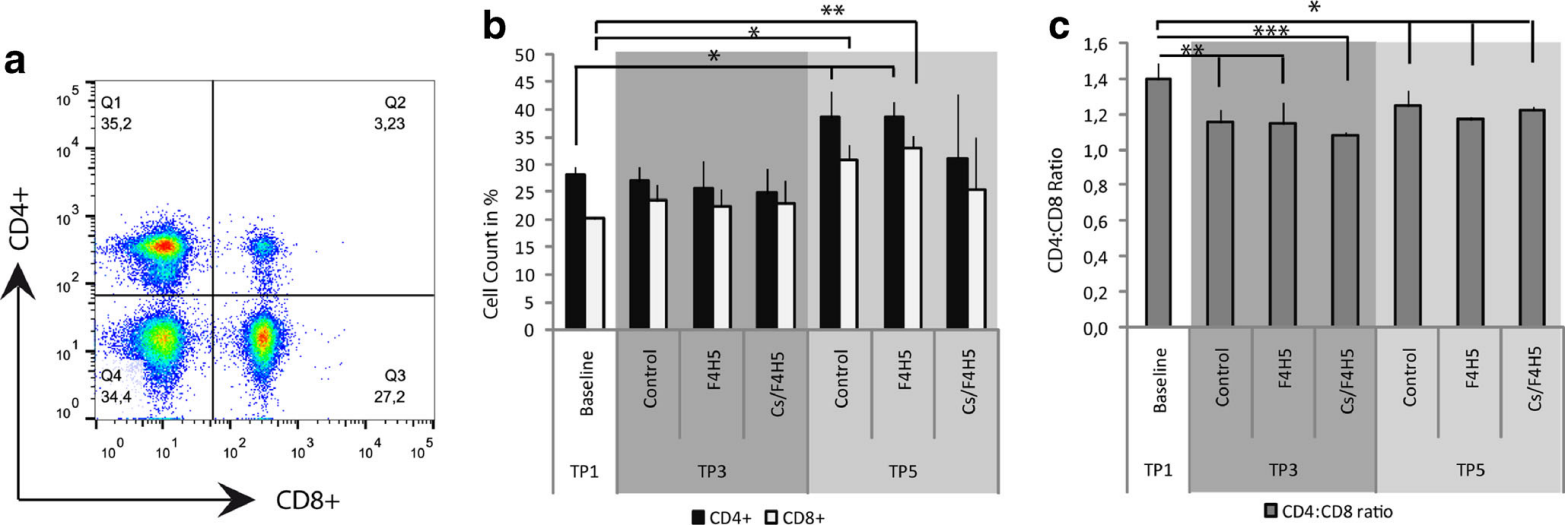
FACS analysis of CD4^+^and CD8^+^T cells of draining lymph nodes after EDE following topical therapy at TP3 and TP5. **a** Representative flow cytometry dot plot. **b** Percentages of CD4^+^ and CD8^+^ cells as proportion of total live cells. At TP5, the total number of CD4^+^ and CD8^+^ cells was increased in control and F4H5 group compared to naïve mice. **c** Calculated CD4:CD8 ratio. CD4:CD8 ratio was significantly reduced compared to baseline (naïve mice). Data representing mean ± SD of *n* = 3 mice/group. Statistics were calculated using ANOVA. *P*-values ≤ 0.05 were considered to be significant (* *p* ≤ 0.05, *** *p* ≤ 0.0001)

FACS analysis was performed in the late-treatment regimen comparing controls with F4H5- and Cs/F4H5-treated groups (Fig. 5). Analysis of CD4^+^ and CD8^+^ lymphocytes from lymph nodes demonstrated no alterations between the groups at TP3 and TP5. Furthermore, no differences were detectable in the percentage of CD4^+^ T cells following 7 days of treatment (TP3) with topical Cs/F4H5 in comparison to control and baseline. At day 35 (TP5), compared to naïve mice, the percentage of CD4^+^ T cells was significantly increased in the control and F4H5 groups, but not in the Cs/F4H5 group (Fig. 5b). In addition, the percentage of CD8^+^ T cells in cervical lymph nodes was increased at TP5 in the control and F4H5 group compared to naïve mice. On TP3 and TP5, the CD4:CD8 T cell ratio was significantly less in all groups in comparison to baseline (Fig. 5c). No differences between the groups were detected.

**Fig. 6:**
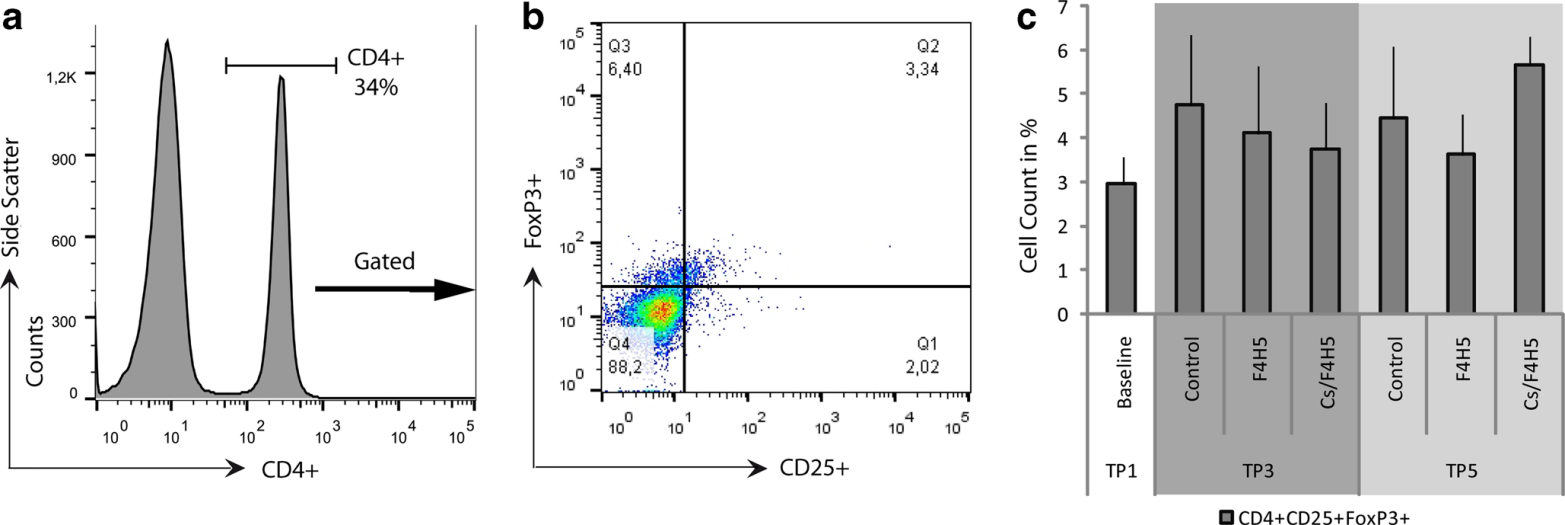
FACS analysis of CD4^+^CD25^+^FoxP3^+^Treg cells of draining lymph nodes after EDE following 7 days of topical therapy (TP3) and after 3 weeks of therapy (TP5). **a** Gating scheme and representative histogram and dot plot graph. **b** Treatment with Cs/F4H5 or F4H5 resulted in no significant differences in the percentage of CD4^+^CD25^+^FoxP3^+^ cells compared to naïve and untreated control mice. Data representing mean ± SD of *n* = 3 mice/group

FACS analysis of CD4^+^CD25^+^FoxP3^+^Tregs of draining lymph nodes resulted in levels between 3 and 6% of cells in draining lymph nodes, with no differences between groups or time points (Fig. 6).

## Discussion

Topical cyclosporine (Cs) is an established immunomodulatory medication indicated for treatment of DED accompanied with inflammation of the ocular surface. It is additionally used in vernal and atopic conjunctivitis, blepharitis, and meibomian gland dysfunction, as well as in LASIK-associated dry eye and ocular graft-versus-host disease [7]. Cs inhibits the activation of T cells and the apoptosis of epithelial cells and reduces proinflammatory cytokines like IL-6. Thereby, Cs clinically decreases corneal staining, increases tearfilm break-up time as well as tear production, and enables patients to decrease their frequency of artificial tear supplement [7].

Cs is a highly lipophilic substance that is typically formulated as emulsions, which often result in side-effects such as burning and stinging sensations [15, 16] in part attributable to the vehicle used [17]. Since the introduction of SFAs, a novel drug carrier system is available that allows to formulate Cs as a preservative- and surfactant-free clear solution. For these reasons, Cs formulated in SFA may be a better tolerable alternative to already available Cs formulations. Furthermore, a solution in combination with the spreading properties of the SFAs might lead to increased delivery of Cs to the site of action.

In our study, scopolamine was steadily applied for 14 days via subcutaneous pumps that together with controlled environmental stress resulted in a reliably dry eye phenotype during acute EDE, even after removal of desiccating stress. Previous studies have shown that Th17 effector T cells maintain the chronic phase of EDE with increased corneal epitheliopathy lasting several weeks after an acute phase of EDE [18]. Therefore, the model used enabled the investigation of the therapeutic effect of Cs/F4H5 in acute as well as in chronic EDE for at least 3 weeks until control groups returned to baseline parameters.

In this study, the therapeutic regimen of 0.05% Cs dissolved in the F4H5 was highly effective in reducing corneal staining and increasing tear production. Compared to the commercially available Cs (Restasis®), Cs/F4H5 demonstrated at least a comparable therapeutic effect, but a significant faster response. Notably, early therapy with Cs/F4H5 starting at day 4 protected mice from developing dry eye, whereas all other groups showed a significant increase of staining compared to baseline. Consistently, this treatment regime was the only one that maintained the number of conjunctival goblet cells in EDE, clearly demonstrating a prophylactic effect of solely Cs/F4H5. No side-effects such as blepharitis, corneal vascularization, etc., were noted in any of the experimental groups.

In a recent phase 1 study with 18 healthy volunteers, repeated applications of Cs/F4H5 (CyclASol®, Novaliq, NCT02113293, http://www.novaliq.de/fileadmin/Downloads/CYS-001_E_final.pdf) have been well tolerated. Hereby, no stinging or burning sensation, irritations, dryness, foreign-body sensation, and no further discomfort of the mucosa or tearing were reported.

A loss of goblet cells (GC) after EDE was described previously, although the level of GCs strongly varied in these studies [13, 19, 20]. In the study presented, the investigation of GC was performed only at the end of the experiment at day 35. Topical Cs was already well known to increase the goblet cell density in murine models of dry eye [5] as well as in in patients [21]. As stated above, early therapy with Cs/F4H5 resulted in a prevention of goblet cell loss in comparison to untreated controls, carrier F4H5, and Restasis®. An effect on goblet cells in the late-treatment regimen was not observed, probably due to a prolonged regeneration phase of goblet cells after initial desiccating stress.

It is known that CD4^+^ T cells play a primary role in the development and progression of dry-eye disease. Desiccating stress leads to infiltration of activated T cells into ocular surface tissues [1]. Such autoreactive CD4^+^ cells are sufficient to induce dry-eye phenotype once adoptively transferred in T-cell-deficient but otherwise healthy nude mice [20]. Since lymph nodes serve as a reservoir for lymphoid cells and are essential for the antigen-presenting cell (APC)-driven activation of autoreactive CD4^+^ T cells [22], draining cervical lymphnodes were investigated in this study. During dry-eye disease, an increase of activated CD69^+^ and CD154^+^ T cells has been reported previously [22, 23]. In the study presented, following 3 weeks of therapy only in the Cs/F4H5 group compared to F4H5 and controls, no increase of CD4+ and CD8+ T-cells was observed, which might explain a potential therapeutic effect of Cs on the regional lymphnode in the late phase of experimental dry-eye disease.

Previous studies [20, 24] further demonstrated that the numbers of CD4^+^CD25^+^FoxP3^+^Tregs play a crucial role in the pathology of dry eye. Specifically, Tregs attenuate effector T cell function and in this way dampen dry eye. Experimentally, a depletion of Tregs led to an exacerbation of adoptively transferred dry-eye disease, whereas the reconstitution with Tregs in athymic mice resulted in a protection against transfer of EDE [20, 24]. Furthermore, it has been described that BALB/c mice, containing a larger pool of Tregs, develop milder EDE than other mice strains, for example C57BL/6 mice [25]. For this reason, the number of Tregs was investigated in this study, but no difference was detected in any of the groups and time points investigated.

This study has some limitations due to its experimental character:

- (i) Desiccating stress was applied for 14 days; this rather long duration might result in metaplasia of the conjunctival and corneal epithelium and consequent impact on the therapeutic effect and readouts, e.g., goblet cell count.
- (ii) In contrast to earlier publications commercial Cs did not show a strong therapeutic effect, which might be due to differences in the experimental setup of desiccating stress [20, 26–30].
- iii. The very recently approved Cs product (Ikervis®) could not be used a control drug, therefore no conclusions can be drawn with this respect.

Therefore, future experiments will also include a shorter desiccating stress period (e.g., 7–9 days) and further controls such as the recently approved Cs product. As all experiments were performed at least twice with sufficient numbers of animals and repeatedly stable clinical phenotypes the setup established is thought to be applicable for further investigations. In addition, pharmacokinetics of F4H5 alone and of the combined product Cs/F4H5 are currently tested in ex-vivo and in-vivo models. These subsequent studies will be supplemented by a phase II clinical trial currently being performed in patients with DED, which tested efficacy and safety profiles of 0.05 and 0.1% Cs/F4H5 in comparison to Restasis® (NCT02617667).

In summary, this experimental study clearly demonstrated a significantly faster and equally effective topical treatment of experimental dry eye using Cs/F4H5 compared to Restasis®. Due to the limitations stated, further experiments will include comparison with other newly available Cs products using a modified protocol of EDE.
